# MicroRNA-103a-3p Promotes Cell Proliferation and Invasion in Non-Small-Cell Lung Cancer Cells through Akt Pathway by Targeting PTEN

**DOI:** 10.1155/2021/7590976

**Published:** 2021-07-07

**Authors:** Haixun Li, Muren Huhe, Jiaxin Lou

**Affiliations:** ^1^Department of Cardiovascular Surgery, The Third Affiliated Hospital of Zunyi Medical University, Zunyi 563000, China; ^2^No. 95948 Units of PLA Hospital, Lanzhou 732750, China; ^3^Inner Mongolia Institute of Digestive Diseases, The Second Affiliated Hospital of Baotou Medical College, Baotou 014030, China

## Abstract

**Background:**

Increasing evidence has suggested that microRNA- (miR-) 103a-3p is crucial for cancer progression. However, the specific mechanism of miR-103a-3p in non-small-cell lung cancer (NSCLC) remains unclear until now. So, it is particularly urgent to clarify the mechanism between them.

**Methods:**

qRT-PCR and western blot were used to measure the expression of miR-103a-3p, PTEN, Akt, and p-Akt. Cell biology experiment was applied to detect the biological function of miR-103a-3p in NSCLC cell lines. Moreover, bioinformatics analysis, luciferase reporter assay, and functional complementation analysis were carried out to investigate the target gene.

**Results:**

miR-103a-3p was highly expressed in primary NSCLC samples and cell lines. miR-103a-3p mimics promoted the proliferation and invasion of NSCLC cells; miR-103a-3p inhibitor had the opposite effect. A double luciferase reporter gene experiment revealed that miR-103a-3p directly targets the PTEN mRNA 3′UTR region. siPTEN inhibited the proliferation and invasion of NSCLC cells. Further mechanistic studies showed that both overexpression of miR-103a-3p and PTEN knockdown reduced the expression of the p-Akt protein. Overexpression of PTEN partially reversed the cancer-promoting effect of miR-103a-3p.

**Conclusion:**

miR-103a-3p promotes the progression of NSCLC via Akt signaling by targeting PTEN, highlighting the role of miR-103a-3p/PTEN/Akt signaling and suggesting miR-103a-3p as a novel therapeutic target for NSCLC.

## 1. Introduction

Non-small-cell lung cancer (NSCLC) has a high fatality rate due to its high recurrence rate [1]. Further investigation is needed for understanding the mechanism of tumorigenesis of NSCLC [2]. Treatment targets for lung cancer also need to be determined [3].

microRNAs are a type of noncoding RNA with lengths of 21–25 nt that can recognize and bind the 3′-untranslated region (UTR) to regulate cell differentiation and development by promoting degradation of the target gene's mRNA, thus inhibiting protein synthesis [4, 5]. In recent years, a large number of miRNAs have been found to regulate lung cancer cell proliferation, apoptosis, and treatment resistance [6–8]. PTEN is a key inhibitor of tumorigenesis [9–11]. Increasing studies have showed multiple miRNAs can regulate the progression of liver cancer [12] and cervical cancer by directly targeting PTEN [13]. However, which miRNAs regulate PTEN in lung cancer is not well understood.

miR-103a-3p is involved in the progress of a variety of cancers [14–17]. However, the report of miR-103a-3p in NSCLC is rare. We aim to clarify the regulation mechanism of miR-103a-3p on the progress of NSCLC.

## 2. Materials and Methods

### 2.1. Bioinformatics Analysis

The data of 706 lung cancer samples were downloaded from the TCGA database (https://xenabrowser.net/) to explore the expression of miR-103a-3p in NSCLC. Two popular public databases, TargetScan and miRDB, were used to predict miRNA target genes.

### 2.2. Cell Lines

BEAS-2B and NSCLC cell lines (A549, NCI-H1299, NCI-H157, and NCI-H1975) human were purchased from the American Type Culture Collection (ATCC, Manassas, VA, USA). HEK293T was purchased from the Stem Cell Bank of the Chinese Academy of Sciences (Shanghai, China). Cells were routinely cultured as per the manufacturers' methods.

### 2.3. Cell Transfection

miR-103a-3p mimics, miR-103a-3p inhibitor, PTEN siRNA, and their control fragments were obtained from GenePharma (Shanghai, China). The PTEN CDS were edited into the pcDNA3.1 vector to construct the overexpression plasmid. Transfections were performed with Lipofectamine™ 3000 Transfection Reagent (Invitrogen, Carlsbad, CA, USA).

### 2.4. qRT-PCR

Total RNA was obtained using TRIzol reagent (Invitrogen). After measuring RNA concentration, cDNA was generated using PrimeScript™ II 1st Strand cDNA Synthesis Kit (TAKARA, Beijing, China). Real-time quantitative polymerase chain reaction (qRT-PCR) was performed using TB Green® Fast qPCR Mix (TAKARA). U6 was used to normalize miR-103a-3p expression, while GAPDH was used to normalize mRNA expression. All primers were listed in Table 1.

### 2.5. Cell Proliferation Assay

The cells were cultured overnight in 96-well plate at 3000 cells per well and then transfected after adherence. 72 h after transfection, 10 *μ*l MTT (5 mg/ml) was added and continually incubated for 4 h at 37°C. The medium was replaced with 150 *μ*l dimethyl sulfoxide. After the formazan solubilized, a spectrophotometer (FLUOstar OPTIMA, BMG, Germany) was used to measure absorbance at 570 nm.

### 2.6. Colony Formation Assay

The transfected cells were plated in 6-well plates at 2000 cells per well and incubated for 10 days. Then, cells were stained using 0.1% crystal violet for 30 min. Images were obtained on Gel Imaging System (Bio-Rad, Hercules, CA, USA).

### 2.7. Transwell Invasion Assay

The transfected cells were plated in the upper chamber (Corning Costar, Lowell, MA, USA) coated with Matrigel in a serum-free medium at 20000 cells per well. The lower chamber was filled with RPMI-1640 complete medium. After incubation for 24 h, the cells on the bottom surface were fixed with 4% polyformaldehyde and stained with 0.1% crystal violet. Images were captured and counted using an inverted microscope.

### 2.8. Western Blot Analysis

Equal amount of protein sample was separated by SDS-PAGE gel and transferred to a PVDF membrane. The PVDF membrane was blocked with 5% nonfat skim milk and incubated with primary antibodies against PTEN, Akt, p-Akt, and GAPDH at 4°C overnight. Then, it was incubated with the secondary antibodies. The bands were measured using ECL blotting detection reagents (Millipore, Burlington, MA, USA).

### 2.9. Luciferase Reporter Assay

The wild-type target gene PTEN (PTEN-WT) and mutant target gene PTEN (PTEN-MUT) luciferase reporter vectors were constructed to perform the luciferase reporter assay. Following transfection of the luciferase reporter vector and miR-103a-3p mimics into HEK293T cells for 36 h, the Dual-Luciferase Assay System (Promega, USA) was applied to measure the luciferase activity of each group.

### 2.10. Statistical Analyses

The SPSS program (version 18.0) was employed for statistical analyses. All data were performed as the mean ± SD. Student's *t*-tests and one-way ANOVA were applied to assess statistical differences. A *p* < 0.05 was statistically significant.

## 3. Results

### 3.1. miR-103a-3p Expression Is Upregulated in NSCLC Samples and Cell Lines

**Figure 1:**
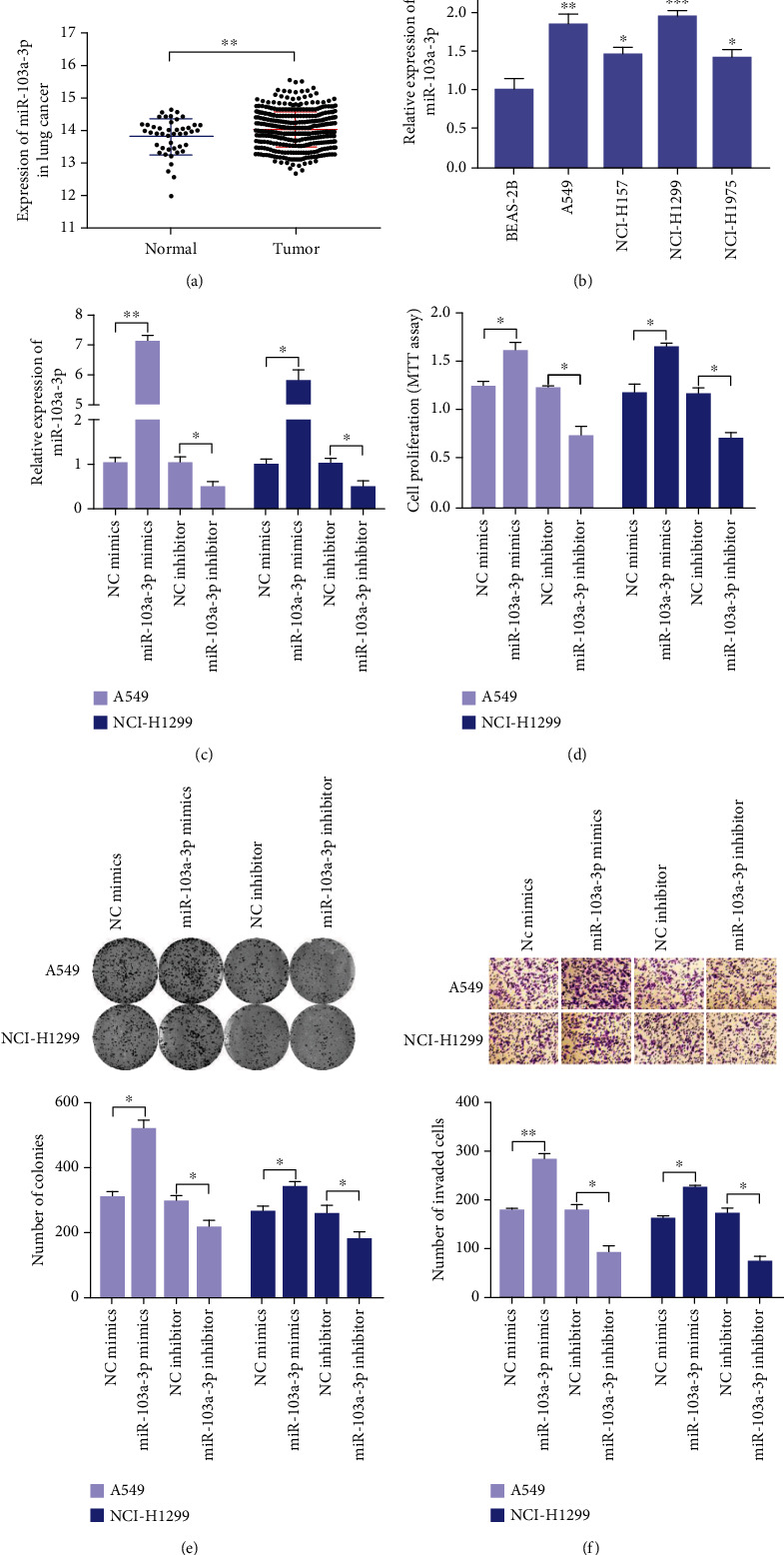
miR-103a-3p promotes NSCLC cell proliferation and invasion. (a) TCGA database analysis of miR-103a-3p expression in NSCLC samples and normal lung samples. (b) Relative expression of miR-103a-3p in NSCLC cell lines (A549, NCI-H157, NCI-H1299, and NCI-H1975) and BEAS-2B were detected by qRT-PCR. A549 and NCI-H1299 cells were transfected with miR-103a-3p mimics or inhibitor for 48 h before the following detections. (c) Relative expression of miR-103a-3p was measured by qRT-PCR. (d) Effect of miR-103a-3p on cell proliferation was examined by MTT assay. (e) Effect of miR-103a-3p on colony-forming ability was detected by the colony formation assay. (f) Effect of miR-103a-3p on invasive capability was assessed by Transwell invasion assay (^∗∗∗^*p* < 0.001, ^∗∗^*p* < 0.01, and ^∗^*p* < 0.05).

To explore the role of miR-103a-3p in NSCLC, we analyzed the data of 706 lung cancer samples from the TCGA database. miR-103a-3p expression was significantly upregulated in NSCLC samples compared with normal lung samples (Figure 1(a)). Furthermore, compared with BEAS-2B, miR-103a-3p expression in NSCLC cell lines was frequently upregulated (Figure 1(b)). The above results suggest that miR-103a-3p may be related to the progression of NSCLC.

### 3.2. miR-103a-3p Promotes the Proliferation and Invasion of NSCLC Cells In Vitro

To investigate the function of miR-103a-3p in NSCLC, miR-103a-3p mimics, miR-103a-3p inhibitor, and their respective controls were transfected into NSCLC cells. qRT-PCR showed that the expression of miR-103a-3p was increased in the miR-103a-3p mimics group and decreased in the miR-103a-3p inhibitor group (Figure 1(c)). The MTT assay results showed that the proliferation of the miR-103a-3p mimic groups was significantly enhanced while that of the miR-103a-3p inhibitor groups was significantly suppressed (Figure 1(d)). The colony formation assay showed that overexpression of miR-103a-3p promoted the colony-forming ability of NSCLC cells (Figure 1(e)). The invasion assay revealed that overexpression of miR-103a-3p enhanced cell invasion, while miR-103a-3p inhibition showed the opposite effects (Figure 1(f)). These results indicate that miR-103a-3p plays an important role in promoting proliferation and invasion of NSCLC cells.

### 3.3. PTEN Is a Direct Target of miR-103a-3p

**Figure 2:**
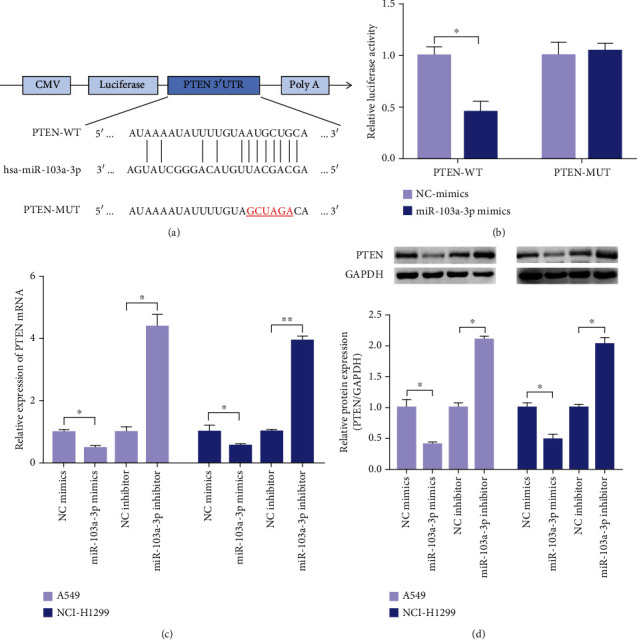
PTEN is a target gene of miR-103a-3p. (a) Sequence diagram of miR-103a-3p with its binding site in PTEN 3′-UTR. (b) Luciferase reporter assay detection for HEK293T transfected with miR-103a-3p mimics or PTEN-WT or PTEN-MUT. Effect of miR-103a-3p on PTEN mRNA (c) and protein (d) expressions was detected by qRT-PCR and western blot (^∗∗^*p* < 0.01, ^∗^*p* < 0.05).

To elucidate the potential mechanism by which miR-103a-3p regulates NSCLC cell proliferation and invasion, we identified the target genes of miR-103a-3p using the TargetScan and miRDB online databases. The tumor suppressor gene PTEN was predicted as a potential target of miR-103a-3p (Figure 2(a)). We performed a luciferase reporter gene assay to validate whether miR-103a-3p directly targets the PTEN 3′-UTR. The luciferase activity of the reporter gene containing wild-type PTEN 3′-UTR was significantly reduced by overexpression of miR-103a-3p, while the luciferase activity of the mutant PTEN 3′-UTR had no significant changes (Figure 2(b)). To clarify whether miR-103a-3p had a direct regulatory effect on PTEN, we transfected miR-103a-3p mimics or inhibitor into lung cancer cells. qRT-PCR showed that the expression of PTEN mRNA was significantly decreased by miR-103a-3p mimics but increased by miR-103a-3p inhibitors (Figure 2(c)). Western blotting showed that the expression of PTEN was decreased in the miR-103a-3p mimic groups and increased in the miR-103a-3p inhibitor groups (Figure 2(d)), consistent with the qRT-PCR results. These data indicate that miR-103a-3p directly bounds to the PTEN 3′-UTR and regulates PTEN expression negatively.

### 3.4. Knockdown of PTEN Can Inhibit Proliferation and Invasion of NSCLC Cells

**Figure 3:**
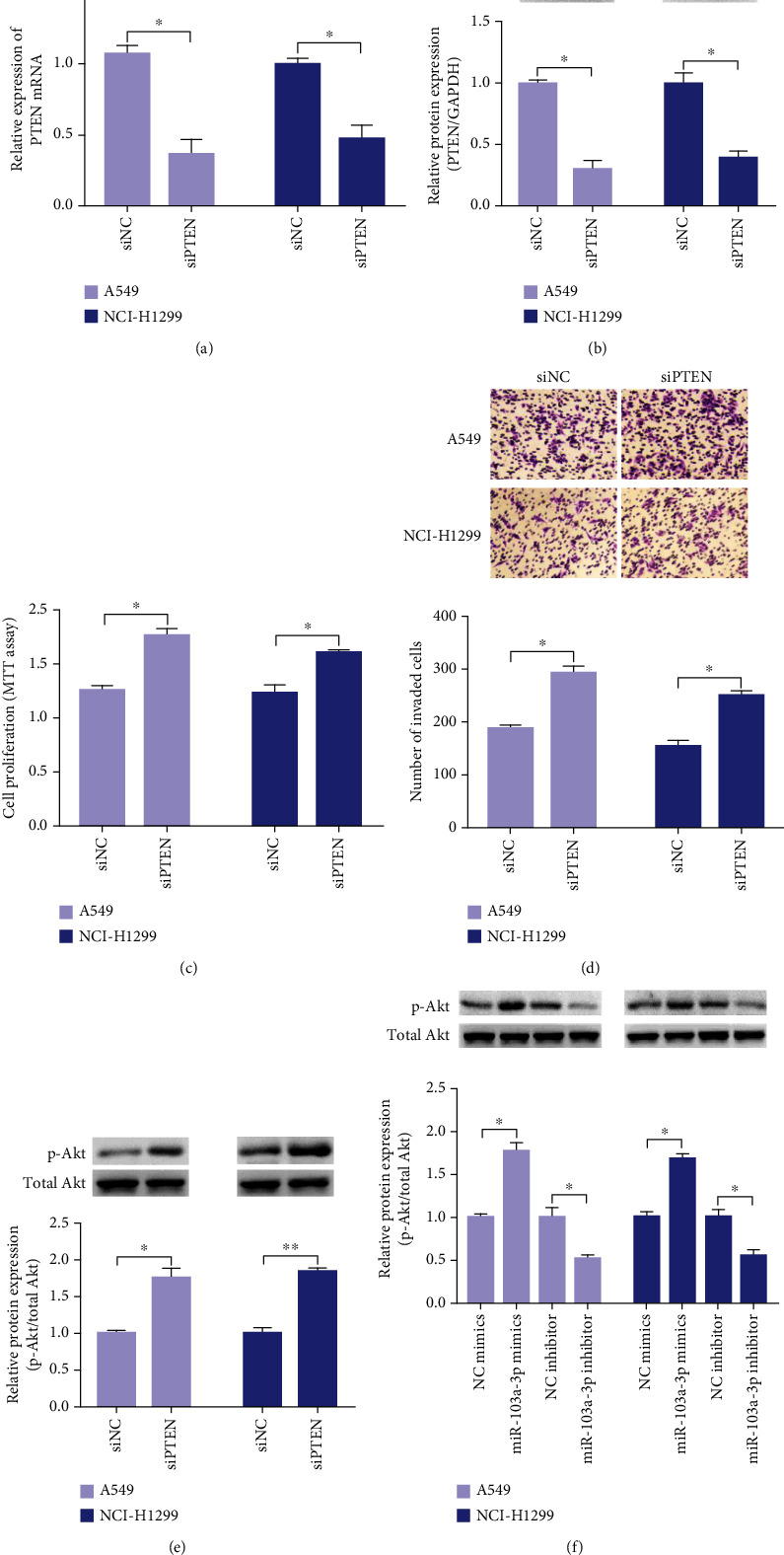
siPTEN promotes the proliferation, invasion, and Akt signaling in NSCLC cells. Relative expression of PTEN mRNA (a) and protein (b) were detected by qRT-PCR and western blot after transfected with PTEN siRNA or NC siRNA for 48 h. (c) MTT assay on effect of PTEN knockdown. (d) Transwell invasion assay on effect of PTEN knockdown. Relative expression of p-Akt/Akt protein was detected by western blot after PTEN knockdown (e) or transfected with miR-103a-3p mimics/inhibitor (f) (^∗∗^*p* < 0.01, ^∗^*p* < 0.05).

To verify whether PTEN exerts a tumor suppressor effect in lung cancer, we inhibited the expression of PTEN in NSCLC cells by transfection of PTEN-specific siRNA. mRNA and protein expressions of PTEN were significantly reduced following transfection of PTEN siRNA (Figures 3(a) and 3(b)). Meanwhile, the proliferation and invasion of lung cancer cells were significantly inhibited (Figures 3(c) and 3(d)). These results suggested that PTEN exerts a tumor suppressor effect in lung cancer.

### 3.5. Knockdown of PTEN Promotes the Activation of Akt Signaling in NSCLC Cells

To investigate the downstream regulatory pathways of PTEN, we detected the expression of Akt phosphorylation in NSCLC cells. Knockdown of PTEN significantly increased the expression of p-Akt, with no significant effect on the total Akt level (Figure 3(e)). Consistently, the reduction of PTEN caused by miR-103a-3p mimics significantly promoted the expression of p-Akt. By contrast, the miR-103a-3p inhibitor significantly suppressed the expression of p-Akt (Figure 3(f)). These results suggest that PTEN and miR-103a-3p are involved in the regulation of Akt signaling in lung cancer cells.

### 3.6. miR-103a-3p Promotes Lung Cancer Cell Proliferation and Invasion by Targeting PTEN

**Figure 4:**
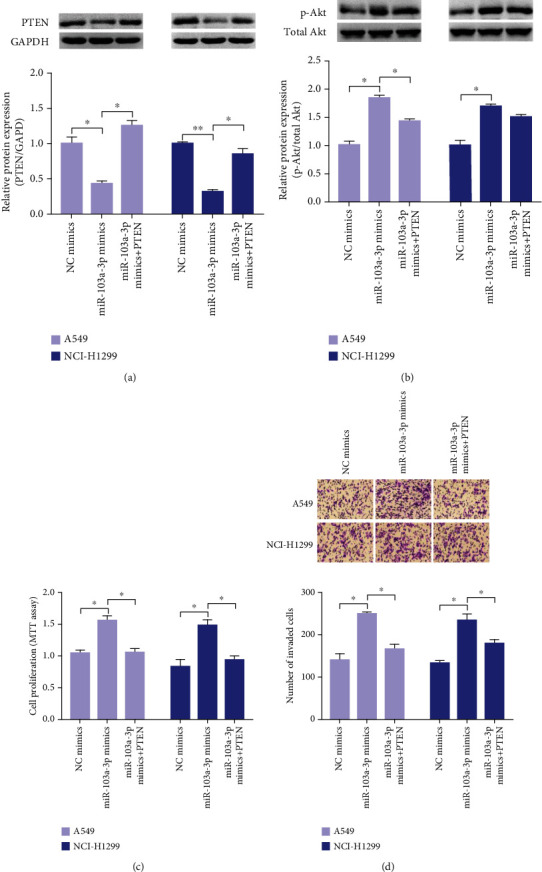
Overexpression of PTEN partly reverses the cancer-promoting effect of miR-103a-3p. A549 and NCI-H1299 cells were cotransfected with miR-103a-3p mimics with or without PTEN expression plasmid for 48 h. Relative expression of PTEN protein (a) and p-Akt/Akt protein (b) was detected by western blot. (c) MTT assay. (d) Transwell invasion assay (^∗∗^*p* < 0.01, ^∗^*p* < 0.05).

To clarify the role of PTEN in miR-103a-3p-promoted proliferation and invasion in NSCLC cells, we performed a rescue experiment. The PTEN expression plasmid and miR-103a-3p mimics were cotransfected into NSCLC cells. The reduction of PTEN protein in miR-103a-3p mimic-transfected cells was significantly restored by transfection of the PTEN overexpression vector (Figure 4(a)) while overexpression of PTEN reduced p-Akt and reversed the promotion of miR-103a-3p overexpression on p-Akt (Figure 4(b)). In addition, the positive effect of miR-103a-3p on lung cancer cell proliferation and invasion was partially reversed by PTEN overexpression (Figures 4(c) and 4(d)). In summary, these results indicate that miR-103a-3p promotes the proliferation and invasion of lung cancer cells by downregulating PTEN.

## 4. Discussion

As a tumor promotor or suppressor, miR-103a-3p can regulate tumor progression in various cancers. Serum miR-103 is a biomarker for the diagnosis of breast cancer [14], while miR-103a-3p inhibits the proliferation of glioma stem cells and functions as a tumor suppressor [15]. miR-103a-3p can also promote gastric cancer cell proliferation by targeting ATF7 [17]. Moreover, miR-103 is upregulated in colorectal cancer, where it promotes the progression of colorectal cancer by targeting DICER and PTEN [18]. In recent studies, miR-103a-3p has been reported to promote migration, invasion, and apoptosis of thyroid cancer cells by downregulating LATS1 via Hippo signaling [19], while in prostate cancer, miR-103a-3p has been shown to suppress the proliferation and invasion by targeting D52 [20]. However, the biological function of miR-103a-3p in NSCLC has not been fully studied. Our study showed that miR-103a-3p is highly expressed in NSCLC samples and cell lines and regulates the proliferation and invasion of NSCLC cells.

miRNAs usually participate in cancer progression at the level of epigenetic regulation. Therefore, we used two bioinformatics online databases to investigate the target genes of miR-103a-3p. PTEN was selected as a target gene for further research because of its role in tumor progression. Expression of PTEN has been shown to be decreased in NSCLC patients, and its deficiency is related to cancer development and progression [21–23]. PTEN can inhibit tumor progression by blocking the PI3K/Akt pathway by positively regulating the phosphorylation of PIP3 at the 3-phosphate site [24–26]. Interestingly, PTEN has recently been studied as a target gene of miRNAs in various cancers. miR-21 suppresses progression of human gastric cancer cells by targeting PTEN/Akt signaling [27]. miR-200a promotes the development of ovarian cancer by targeting PTEN [28]. miR-106b and miR-93 promote breast cancer cell proliferation and invasion by suppression of PTEN via PI3K/Akt [29]. By using luciferase reporter gene assays, qRT-PCR, and western blot, we proved that PTEN is a downstream target gene of miR-103a-3p in NSCLC. In addition, expression of p-Akt was increased by transfected with miR-103a-3p mimics in NSCLC cells. Knockdown of PTEN showed similar functions as miR-103a-3p mimics. Overexpression of PTEN could partially reverse the effect of miR-103a-3p on NSCLC cells. These results indicate that miR-103a-3p exerts its biological functions by directly targeting PTEN.

In conclusion, our study demonstrates the tumor promotion effect of miR-103a-3p in NSCLC. miR-103a-3p can promote the proliferation and invasion of NSCLC cells by targeting PTEN via inhibition of Akt signaling. Our study provides a new perspective into the miR-103a-3p-mediated NSCLC progression, which may be a potential therapeutic target for NSCLC in the future.

## Figures and Tables

**Table 1:** Primers and oligonucleotides used in this study.

Name	Sequence (5′-3′)
miR-103a-3p RT	GTCGTATCCAGTGCGTGTCGTGGAGTCGGCAATTGCACTGGATACGACTCATAGC
miR-103a-3p-forward	ATCCAGTGCGTGTCGTG
miR-103a-3p-reverse	TGCTAGCAGCATTGTACAGG
U6-RT	CGCTTCACGAATTTGCGTGTCAT
U6-forward	GCTTCGGCAGCACATATACTAAAAT
U6-reverse	CGCTTCACGAATTTGCGTGTCAT
miR-103a-3p mimics	AGCAGCAUUGUACAGGGCUAUGA
NC mimics	ACGGUUAGACCGAUUCCGAAUCCGCG
miR-103a-3p inhibitor	TCATAGCCCTGTACAATGCTGCT
NC inhibitor	CAGTACTTTTGTGTAGTACAA
PTEN-forward	ACCAGTGGCACTGTTGTTTCAC
PTEN-reverse	TTCCTCTGGTCCTGGTATGAAG
GAPDH-forward	AGGTCCACCACTGACACGTT
GAPDH-reverse	GCCTCAAGATCATCAGCAAT
siNC-sense	UUCUCCGAACGUGUCACGUTT
siNC-antisense	ACGUGACACGUUCGGAGAATT
siPTEN-sense	GGUGUAAUGAUAUGUGCAUTT
siPTEN-antisense	CAAAUUUAAUUGCAGAGUUTT

## Data Availability

The data used to support the findings of this study are available from the corresponding author upon request.
